# Shifting syndromes: Sex chromosome variations and intersex classifications

**DOI:** 10.1177/0306312718757081

**Published:** 2018-02-09

**Authors:** David Andrew Griffiths

**Affiliations:** Department of Sociology, University of Surrey, Guildford, UK

**Keywords:** classification, DSD, intersex, Klinefelter’s syndrome, sex chromosomes, turner syndrome

## Abstract

The 2006 ‘Consensus statement on management of intersex disorders’ recommended moving to a new classification of intersex variations, framed in terms of ‘disorders of sex development’ or DSD. Part of the rationale for this change was to move away from associations with gender, and to increase clarity by grounding the classification system in genetics. While the medical community has largely accepted the move, some individuals from intersex activist communities have condemned it. In addition, people both inside and outside the medical community have disagreed about what should be covered by the classification system, in particular whether sex chromosome variations and the related diagnoses of Turner and Klinefelter’s syndromes should be included. This article explores initial descriptions of Turner and Klinefelter’s syndromes and their subsequent inclusion in intersex classifications, which were increasingly grounded in scientific understandings of sex chromosomes that emerged in the 1950s. The article questions the current drive to stabilize and ‘sort out’ intersex classifications through a grounding in genetics. Alternative social and historical definitions of intersex – such as those proposed by the intersex activists – have the potential to do more justice to the lived experience of those affected by such classifications and their consequences.

## Introduction

The classification of bodily variations is a complex, messy process. Classifications of variations of sex characteristics, or intersex traits, have changed significantly throughout history. In general, *intersex* refers to the state of being born with biological sex characteristics that vary from what is typically thought of as exclusively male or female. In 2005, a meeting of mainly medical professionals proposed a new classification system, one that would move away from taxonomies of ‘hermaphroditism’ and the terminology of intersex, to the new term, ‘disorders of sex development’ or DSD. The following year, the ‘Consensus statement on management of intersex disorders’ was published, in which DSDs were defined as ‘congenital conditions in which development of chromosomal, gonadal, or anatomical sex is atypical’ (Hughes et al., 2006; Lee et al., 2006). The DSD classification was an attempt at ‘sorting things out’ (Bowker and Star, 1999), in part through a grounding of the classification system in genetic science. I argue that this is very much a ‘promissory science’ (Hedgecoe, 2004), built upon the expectation that, at some time in the future, improved genetic testing and increased genetic understandings will lead to clearer classification, quicker diagnosis and better treatments. Can individuals affected by these diagnoses or classifications expect such improvements through this DSD framework and the promise of a clear genetics of intersex *to come*?

In this article, I focus on the history of inclusion and exclusion of Turner and Klinefelter’s syndromes within intersex classifications, to explore the consequences for classification systems and the individuals named by them. At different historical moments, these chromosome variations have been central to intersex classifications, peripheral to them, or even excluded. While Turner and Klinefelter’s syndromes are often discussed in scholarship on intersex, the question of their inclusion or exclusion in intersex taxonomies is often discussed only in the context of the question: How many intersex people are there? In this article I go beyond this question, critically analysing the inclusion or exclusion of sex chromosome variations in historical and contemporary taxonomies of intersex/DSD. I offer this historical account of biomedical classifications to elucidate contemporary concerns and controversies, and as such am concerned with both the practice and politics of science, as an example of what has been described as the ‘engaged programme’ (Sismondo, 2008), or the ‘reconstructivist agenda’ in science and technology studies (Woodhouse et al., 2002). This approach seeks to be both theoretical and practical, using the tools of constructivism to ask normative questions about what could be considered ‘better’ in technoscientific theory and practice, including working to produce ‘results of political or practical value for promoting democratic control of and participation in science and technology’ (Sismondo, 2008: 20). That is, the engaged or reconstructivist approach ‘assumes that technoscience is contingent and socially negotiated – and goes on to tackle the problems of how to reconstruct technoscience to promote a more democratic, environmentally sustainable, socially just, or otherwise preferable civilization’ (Woodhouse et al., 2002: 297–298).

As Bowker and Star explain, classifications always involve social and ethical choices: ‘Each standard and category valorizes some point of view and silences another. This is not inherently a bad thing – indeed it is inescapable. But it *is* an ethical choice, and as such it is dangerous – not bad, but dangerous’ (Bowker and Star, 1999: 5–6, emphasis in original). Furthermore, ‘people (and the information systems they build) routinely conflate formal and informal, prototypical and Aristotelian aspects of classification. There is no such thing as an unambiguous, uniform classification system’ (p. 322).^1^ The consensus statement structures its ‘example of a DSD classification’ around Aristotelian ideals of classification; that is, each variation can be placed into mutually exclusive, non-ambiguous, categories based on necessary and sufficient properties. In this case, the classification categories are structured around the karyotype (features of the chromosomes in the nucleus of the cell, including number, size and shape): ‘Sex chromosome DSD’; ‘46,XY DSD’; and ‘46,XX DSD’. Within these categories, individual diagnoses are further classified, based on hormonal, gonadal, chromosomal or other bodily variations or differences. In practice, however, the ideal of an Aristotelian classification system ‘gives way’ to ‘fuzzier’ classification systems populated by ‘heterogeneous objects linked by metaphor or analogy’ (Bowker and Star, 1999: 65). Fuzzy or prototypical classification suggests that some variations can be ‘more like’ DSDs that others, based on certain prototypical features of the DSD classification system.

The DSD classification was quickly adopted by the medical profession. Some scholars of intersex from the humanities and social sciences were also supportive of the shift (Feder, 2009; Koyama, 2006). However, many scholars were critical of defining bodily variations as disordered (Davis, 2011; Holmes, 2011; Reis, 2007). There were also a range of views on the new terminology within support, advocacy and activist organizations. Some groups adopted a pragmatic approach, employing the DSD language so as to engage directly with medical knowledge and provide medical advice (e.g. dsdfamilies, 2017). Other individuals and organisations rejected the move to DSD, arguing that to refer to bodily variations as ‘disorders’ is inherently pathologizing (e.g. Organisation Intersex International in the United Kingdom (OII-UK), 2014). Moving from intersex to DSD represents a series of scientific, medical, normative and ethical choices, choices that have real effects on the individuals represented in the classification systems. Acceptance or non-acceptance of the DSD terminology has profound effects on individuals described by this language, including on their self-perception, access to medical care, and relationships with medical professionals and with family members (Davis, 2015). Despite the fact that the consensus statement explicitly mentions the importance of psychosexual outcomes and support, the focus on promissory genetics has led to a focus on biomedical intervention rather than on psychosocial support (Liao et al., 2015). The Chicago meeting was attended by only two non-medical experts, and a large amount of DSD research has since been directed towards molecular and genetic knowledge production within large data banks (Ahmed et al., 2011).

In this article, I investigate how sex chromosome variations have been defined differently at different times. I focus on their inclusion in or exclusion from classifications of intersex and the practical implications for individuals with these variations. Turner syndrome was first described in 1938 and Klinefelter’s syndrome in 1942. Initially, these syndromes were descriptions of an association of symptoms in an individual body. As karyotype possibilities beyond XX or XY were described in the late 1950s and early 1960s, and as human medical genetics was being transformed ‘from a medical backwater to an appealing medical research frontier between 1955 and 1975’ (Lindee, 2005: 1), these syndromes became genetic ‘sex-reversals’ with doubt also cast upon gender and sexuality. While current conventional understandings of genetics are likely to focus at a smaller level than the chromosome (the recent DSD classification system goes beyond karyotype to consider the role of individual genes), during this historical period the karyotype was synonymous with ‘genetic sex’.

As Mol (2002: 5) argues, bodies, scientific knowledges, technologies and classifications are always multiple, coming into being or ‘enacted’, along with the practices with which they are described. Mol prefers ‘enact’ over the perhaps more familiar metaphor of performance, to avoid associations with ideas of a ‘real reality’ somewhere ‘backstage’, as well as ideas of a difficult accomplishment, or associations with performative effects beyond the moment (p. 32). Enacting ‘suggests that activities take place – but leaves the actors vague’, and it is possible to say that ‘in the act, and only then and there, something *is* – being enacted’ (p. 33, emphasis in original). Objects (including bodies and classification systems) are enacted in specific local practices, and thus become ‘heavy with meaning’, a *meaning* that has been attributed. Such attributions have a history, and they are culturally specific, opening them up for historical and social scientific investigation’ (p. 10).

I do not wish to argue that there is a ‘truth’ of either Turner or Klinefelter’s syndromes that different classification systems approach, but rather that classification systems are historically and culturally situated, enact sex chromosomes and bodies in different ways, and are thus open to critical investigation. I follow Roberts’s (2007) work on ‘sex hormones’ to offer an analysis of the sexed body through the history of sex chromosome variations, as part of the reconstructive work of feminist technoscience studies. As she argues, this is one of the key ‘roles’ of feminist work – to critically analyse biological and scientific frameworks but also to develop and construct new concepts and frameworks for understanding the biological and the social (Roberts, 2007: 23).

The stakes of this investigation are numerous, but I am particularly interested in three distinct issues. First, there are stakes for individuals named by diagnoses, taxonomies and classification systems. Scholarship on intersex has shown that individuals have different medical and non-medical experiences based on whether they accept DSD terminology. The description of symptoms, syndromes, genetic conditions or disorders is not just an issue of meanings; bodily and social realities shift depending on the meaning-making practice of classification. Second, there are stakes for medicine. As I will show, the efficacy and worth of the DSD classification system for medicine rests upon inclusion or exclusion of sex chromosome variations. The value of the system for medicine derives from its being grounded in genetics and the promissory, not in genitals and the assignment of gender as close to the moment of birth as possible; this grounding in genetics demands that sex chromosome variations are central to the DSD classification. And finally, there are wider stakes for democratic participation in practices of biomedical science, particularly around questions raised by intersex human-rights activism that foreground the power relations that prioritize genetic and molecular knowledge over lived experience. Whether Turner or Klinefelter’s syndromes are included or excluded from intersex classifications is not just an argument about numbers of intersex people. Rather, it is part of a broader argument about who gets to name what in biomedicine, and how scientific power can be appropriated and/or resisted through refusing classifications, or building alliances around alternatives.

## ‘Disorders of sex development’

Recent debates about how many intersex people there are reveal how contested the category has been, and the centrality of Turner and Klinefelter’s syndromes to that question. In 2000, Anne Fausto-Sterling claimed a 1.7% incidence; this was based on a review of medical literature over a roughly 50-year period and suggested as ‘an order-of-magnitude estimate rather than a precise count’ (Fausto-Sterling, 2000: 51; see also Blackless et al., 2000). Fausto-Sterling (2000) defined intersex as the phenomenon of bodies that exhibit a mix of ‘anatomical components conventionally attributed to both males and females’ (p. 31). Elsewhere, she defined an intersex person as an ‘individual who deviates from the Platonic ideal of physical dimorphism at the chromosomal, genital, gonadal, or hormonal levels’ (Blackless et al., 2000: 161). Fausto-Sterling’s estimate included Turner and Klinefelter’s syndromes. Two years later, after Fausto-Sterling’s estimate was published, psychologist Leonard Sax disputed her figures, narrowing the definition of intersex to individuals ‘who have XY chromosomes with predominantly female anatomy, XX chromosomes with predominantly male anatomy, or ambiguous or mixed genitalia’ (Sax, 2002: 174). Significantly, Sax did not consider either Klinefelter’s or Turner syndrome as intersex, as they are not ‘associated with ambiguous genitalia, or with any confusion regarding sexual identity’ (177). Whilst Fausto-Sterling’s broader system was organized around multiple kinds of variation from ‘ideals’ of dimorphism, Sax’s suggested taxonomy performs boundary management by emphasizing anatomy that does not match either XX or XY karyotypes and genital ‘ambiguity’ as the prototypical traits of intersex, and excluding sex chromosome variations beyond XX/XY where these traits are not in evidence.

This lack of consensus informed the introduction of the DSD taxonomy. In 2005, Alice Dreger, Cheryl Chase (the founder of the Intersex Society of North America), and three medical professionals published an article in the *Journal of Pediatric Endocrinology and Metabolism* arguing for a change in clinician’s nomenclature and taxonomy. Significantly, they argued that intersex classifications do not represent natural kinds, but rather constitute practical taxonomies (Dreger et al., 2005: 730). Clinicians had previously followed a taxonomy divided into: 1. Females; 2. Males; 3. Female pseudohermaphrodites (some male, mixed or ambiguous sex characteristics present with ovaries and XX chromosomes); 4. Male pseudohermaphrodites (some female, mixed or ambiguous sex characteristics present with testes and XY chromosomes); and 5. True hermaphrodites (presence of an ovotestis, or both a testis and an ovary). This classification system, based on the tissue structure of the gonads, is referred to as the Klebs taxonomy or Klebs system, after 19th century pathologist Theodor Albrecht Edwin Klebs (Dreger, 1998). Dreger et al.’s paper questioned both the scientific value and practical clinical benefit of basing classifications in gonadal differences, suggesting instead a practice of classifying individual specific diagnoses under an umbrella term of ‘disorders of sexual differentiation’.

Later in 2005, Chase participated in a meeting on intersex in Chicago, jointly organised by Dr Peter Lee of the US-based Lawson Wilkins Pediatric Endocrine Society and Dr Ieuan Hughes of the European Society for Paediatric Endocrinology. The meeting was attended by 50 experts, only two of whom (Chase included) were not medical professionals. The suggestion of a nomenclature change to ‘disorders of sexual differentiation’ was raised (Feder, 2014). In Chase’s own account, she wanted ‘a language that is really appealing to doctors, that fits somehow into the way they think about things, so it’ll be easy to adopt’ (Davis, 2015: 45). In other words, what was needed was a pragmatic classification system for enacting variations of sex characteristics in a way that was familiar to the medical profession. Chase also stated that she wanted the classification ‘not to have the word “pseudo”’, ‘not to have any indication of sex or gender’ and ‘not to have the word “hermaphrodite” or “true”‘ (Davis, 2015: 45). In Chase’s words, ‘then they went off and they decided there were some technical reasons why they preferred “development” to “differentiation”, and they got it adopted’ (Davis, 2015: 45).

The ‘Consensus Statement on Management of Intersex Disorders’ was published the following year, in 2006. The change to a DSD taxonomy was justified as follows:

Terms such as intersex, pseudohermaphroditism, hermaphroditism, sex reversal, and gender based diagnostic labels are particularly controversial. These terms are perceived as potentially pejorative by patients, and can be confusing to practitioners and parents alike. The term ‘disorders of sex development’ (DSD) is proposed, as defined by congenital conditions in which development of chromosomal, gonadal, or anatomical sex is atypical. (Hughes et al., 2006: 554)

The consensus statement therefore followed Dreger and Chase’s suggestions, particularly in moving away from the hierarchical categorization of pseudo and true hermaphroditism, and in broadening the medical classification system beyond the gonads.

The 2006 consensus statement includes ‘45,X (Turner syndrome and variants)’ and ‘47,XXY (Klinefelter syndrome and variants)’ as ‘Sex Chromosome DSD’. However, discussion about these variations is limited and estimates of intersex in the consensus statement are reserved for a statement that ‘genital anomalies occur in 1 in 4500 births’ (Hughes et al., 2006: 554). There is also no consensus in medicine as to the boundaries of the DSD classification system. Despite the ‘consensus’ to include Turner and Klinefelter’s syndromes in DSD, some medical professionals have suggested they be excluded (Aaronson and Aaronson, 2010; Wit et al., 2007), and that the features that anchor the DSD should necessarily be gonads, genitals and the assignment of gender as close to the moment of birth as possible. I will return to these points. Crucially, the DSD classification rests upon the choice to include these syndromes, a choice that, as Bowker and Star (1999) point out, is not merely conceptual, but also normative and ethical, and that has clear consequences for the people so classified.

Some individuals and organizations have been critical of the shift to DSD terminology; many organizations used the term ‘intersex’ to reframe variations in sex characteristics as a social issue, not a medical emergency. The term ‘disorder’ structures the classification around the normal and the abnormal – a fact implicitly recognized by medical professionals when they suggested using ‘differences’ or ‘variations’ (rather than ‘disorders’) of sex development when speaking with individuals and family members (Ahmed et el., 2011). At the World Professional Health Association for Transgender Health conference in 2016, representatives of a number of global intersex activist communities presented evidence of ongoing medical mistreatment of intersex individuals internationally. As part of a presentation, Miriam van der Have, co-chair of Organisation Intersex International Europe (OII-Europe) and chair of Nederlands Netwerk Intersekse/DSD suggested a shift in the definition of the term intersex. Her ‘post-medicine definition’ defines intersex as ‘the lived experience of the socio-cultural consequences of being born with a body that does not fit with normative social constructions of male and female’ (Van der Have, 2016; see also Van der Have, 2017; Van Heesch, 2016). This post-medicine (and by extension, post-DSD) definition, to which I will return, shifts the focus from individual bodily differences, whether at the level of genes, chromosomes, gonads or genitals, to the social context in which such classifications are made, and the ramifications that inclusion or exclusion from normative social classifications can have on individuals. There is a lack of consensus, inside and outside medicine, as to whether to use the DSD classification system, and if so, what to include and what the central features of the classification are. This is not a new phenomenon.

## Turner and Klinefelter’s syndromes

I turn to the original descriptions of Turner and Klinefelter’s syndromes, to historicize the difficult business of ‘sorting out’ intersex classifications. In 1938, Henry H Turner first described what would become known as Turner syndrome. Turner was an endocrinologist running his own practice and working as assistant professor of medicine and as consulting endocrinologist at the University of Oklahoma’s College of Medicine. He thus saw a large number of individuals with developmental variations. Turner (1938) initially described seven white women aged 15-23, with three sets of symptoms described as ‘retardation in growth and sexual underdevelopment’, ‘slight to marked’ webbing of the skin of the neck without absence or fusion of the vertebrae, and a ‘constantly present’ ‘deformity’ of the elbow (pp. 573–574). These symptoms on their own were not necessarily uncommon; however, he argued that the co-occurrence of all three was rare and could not be recognized by any previous diagnosis.

In 1942, Harry Klinefelter, along with the endocrinologists Fuller Albright and Edward Reifenstein, described what would become known as Klinefelter’s syndrome (Klinefelter et al., 1942). Klinefelter (1986) was a rheumatologist and endocrinologist at the Massachusetts General Hospital in Boston working under the supervision of Albright considered by many the ‘most outstanding clinical endocrinologist in the world’ (p. 1090). Like Turner syndrome, Klinefelter’s syndrome was an association of symptoms: gynecomastia (male breast enlargement), smaller than average testes with aspermatogenesis but without a-leydigism (non-production of sperm but with secretion of Leydig cells, the cells in testes that produce testosterone), and increased excretion of follicle-stimulating hormone (a hormone that plays a role in the regulation of growth, puberty and reproduction). Klinefelter (1942) later stated that while he worked with Albright on the series of nine individuals with these symptoms, it was ‘really just another of Dr. Albright’s diseases’, but that Albright had been charitable in letting him be named first on the 1942 paper (p. 1090). Indeed, in the early 1950s there are references to the syndrome as ‘the K.R.A. syndrome’ (Sougin-Mibashan and Jackson, 1953), after the three authors of the paper. After the initial description, the classification of Klinefelter’s syndrome loosened to include individuals without gynecomastia, but with low testosterone and small testes (Heller and Nelson, 1945; Howard et al., 1950). Here, then, it is adult bodies that can be diagnosed with Turner or Klinefelter’s syndrome, through the association or co-existence of a number of specific bodily symptoms.

The initial description of Turner and Klinefelter’s syndromes illustrate what Jutel (2009: 230) has called the ‘classificatory project’ in medicine. Jutel (2009) identifies a shift in eighteenth-century medicine away from a focus on individual symptoms towards a focus on ‘groups and patterns of symptoms that doctors could reliably recognise’ (p. 281). She draws on Foucault (1973) to suggest that between the late eighteenth and late nineteenth centuries, clinical practice ‘removed the symptom from its previously supreme position, seeing it instead as simply one element in a symptom cluster which would constitute the disease’ (Jutel, 2009: 281). The classificatory project saw a shift from the idea of bodies exhibiting single symptoms to bodies ‘having’ syndromes, evidenced by the association or co-existence of a number of symptoms. This gave new meaning to syndromes as diagnostic categories and shifted the meanings of human bodies as described by diagnosis. The shifts in the classificatory project were also shifts in the different ways that bodies could be enacted by biomedical science.

While the definition of Turner’s and Klinefelter’s as syndromes made up new categories of people and gave classificatory meaning to particular symptom clusters, these categories bore no relationship yet to older categories of hermaphroditism or the emerging category of ‘intersex’. Despite the reproductive issues and effects on sex characteristics associated with these two syndromes, their original descriptions did not suggest that these bodies were in any way ‘in doubt’, with regards to gender and sexuality (Reis, 2009). Original work on Turner syndrome included only women, but a number of published articles identified similar symptom clusters in male individuals (Flavell, 1943; Greenblatt and Nieburgs, 1948; McCullagh, 1948; Membrives et al., 1949; Sougin-Mibashan and Jackson, 1953). Original work on Klinefelter’s syndrome included only men. Significantly, Klinefelter (1986) always maintained that individuals with his syndrome cluster were unproblematically phenotypic males ‘and should never be considered otherwise’ (p. 1092). However, genetic science in the 1950s would significantly change the classification of individuals with diagnoses of Turner or Klinefelter’s syndromes.

## *Drosophila* and Barr bodies

Sex chromosome variations would be central to developments in intersex classifications, and to the development of what Richardson (2013: 104–105) has called the ‘hyperbinary’ view of sex chromosomes. In the 1920s,

the new science of sex, structured around the sex hormone, was firmly established, institutionally and intellectually. An expectation of a dynamically interacting two-tiered structure of explanation for sex at the genetic and hormonal levels fuelled new biological models of sex and irresistibly pulled theorists toward the consistent and parallel terminology of ‘sex hormones’ and ‘sex chromosomes’ (Richardson, 2013: 70; see also Oudshoorn, 1994).

In the first half of the twentieth century, the concept of a sex chromosome was becoming increasingly established. However, the general view was that the X chromosome was determinative of biological sex, and the Y was not determinative, or was even inert (Richardson, 2013: 72). Both scientific and popular discourse until the late 1950s assumed an XY chromosome complement for all men, and XX for all women.

Initially, human sex chromosomes were interpreted in the light of studies of the fruit fly *Drosophila melanogaster*.^2^ *Drosophila* studies began in the early twentieth century (see Harper, 2006). *Drosophila* chromosomes were easy to study, as they were larger, divided rapidly, and fruit flies were easy to obtain. Human sex chromosomes, however, were difficult to study; they are more numerous, smaller, and appropriate cellular material was difficult to obtain. *Drosophila* research in the 1920s had discovered that the X chromosome is female-determinative in the fruit fly; sex is determined by the ratio of X chromosomes to autosomes (chromosomes not associated with sex-determination), with more X chromosomes producing female fruit flies (Richardson, 2013: 105). The discovery of the existence of male flies with XO sex chromosomes, and female flies with XXY supported this theory, and it was assumed that chromosomal sex-determination in humans proceeded in much the same way (Richardson, 2013: 72).

During the first half of the twentieth century, most human genetic research was carried out on male gametes. Sperm cells carry only an X or a Y. This, and the assumptions that the sperm with an X chromosome always produces a female and that male individuals inherit their X from their mother, coupled with the pervasiveness of *Drosophila* research, led to the hyperbinary view that femaleness resided in the X and maleness in the Y chromosome. This was the background against which Canadian researchers Murray Barr and Ewart Bertram made the accidental discovery in 1948 of a small body in the nucleuses of the neurons of cats, while they were studying the neuronal basis of prolonged fatigue in London, Ontario (Harper, 2006: 18). These small bodies were readily identifiable through a routine tissue-staining method. Barr and Bertram realized that this small body was only present in the neurons of female cats, and concluded that the ‘maleness’ or ‘femaleness’ of cells – ‘so far as chromosome content is concerned’ – could be detected with ‘no more elaborate equipment than a compound microscope following staining of the tissue’ (Barr and Bertram, 1949: 163). They published their findings the following year in *Nature*, describing the body as a ‘nucleolar satellite’. This ‘satellite’ became known as the ‘sex chromatin’ – so-named because of colour-staining during identification (Graham and Barr, 1952), and later the ‘Barr body’ (Sohval and Casselman, 1961). This discovery led to the development of better technologies and techniques for studying chromosomes, and sex chromosomes in particular. Chief among these techniques was the ‘buccal smear’, an inexpensive and relatively non-invasive procedure of swabbing cells from the inside of the cheek. These cells could then be stained and ‘smeared’, making easier the identification of the existence of a Barr-body (described as ‘chromatin-positive’) or non-existence (‘chromatin-negative’), and thus the assumed identification of female or male sex.

Barr et al. developed the concept of the sex chromatin by focusing on humans with sex chromosome variations, including those who were beginning to be classified as ‘intersex’. By the 1950s, the clinical ‘management’ of individuals with atypical sex anatomies was increasingly seen as a pressing problem. The Klebs gonad-based classification of individual diagnoses continued, but in practice clinicians often selected the ‘best sex’ rather that the ‘true sex’ to assign an individual (Dreger, 1998; Hird and Germon, 2001; Mak, 2012; Preves, 2003). In part, this was because of the practical clinical difficulty of identifying the ‘truth’ of the gonads. The 1950s was a significant decade for the history of intersex, as it was then that John Money et al. began publishing work on what came to be known as the optimum gender of rearing, or early surgery paradigm.^3^

Prior to the publication of Money et al.’s work, Barr had already begun to align clinical medicine’s concerns around variations of sex characteristics with the genetics of sex (at the time focused at the level of the chromosome). With Margaret Graham and Keith Moore, he argued that the disjunction between medical theory and clinical practice was a pressing problem. They stated that cases of ‘hermaphroditism’ were a tragedy and a ‘developmental error’ that demanded correction through medical action, informed by the ability to test for Barr bodies (Moore et al., 1977 [1953]) were invested in what Cynthia Kraus has called ‘tooling sex’, that is ‘*making* sex an object of research and knowledge’, and an experimental tool, rather than considering sex as an essential ‘natural fact’ (Kraus, 2000: 169, emphasis in original). Moore et al. (1977 [1953]: 109) invested hope in the practical value of the test on the assumption that ‘the chromosomal sex will prove to be a reliable indication of the *dominant* sex of the patient as a whole’. They also emphasized the practical nature of shifting focus from a gonad-based classification to one with more focus on the chromosomes: ‘Such a development would be the more valuable since the dominant sex could be detected *in infancy* by a relatively simple procedure’ (Moore et al., 1977 [1953]: 109, emphasis in original). Chromosomes offered a narrative of hyperbinary sex essentialism for intersex classification. Importantly, the Barr body test offered a practical, simple and inexpensive clinical tool, for ‘sorting things out’ when clinicians were faced with an individual they could not easily classify within the binary male-female system.

## ‘Sex-reversals’

The classification of individuals based on karyotypes, as identified by presence or absence of Barr bodies, led to the assumption that individuals with Turner and Klinefelter’s syndromes were ‘sex-reversals’ (Lennox et al., 1958: 126). Within the hyperbinary view of chromosomal sex, ‘sex-reversals’ referred to individuals with typically male bodies but XX karyotype, or individuals with typically female bodies but XY karyotype. In 1954, geneticist and paediatrician Paul Polani et al. published ‘Chromosomal sex in Turner’s syndrome with coarctation of the aorta’ in the *Lancet*. As coarctation (narrowing) of the aorta (the main artery of the body, which travels from the heart to the abdomen) is most commonly described in male individuals, Polani et al. (1954) began to wonder ‘whether there is accurate proof that patients with Turner’s syndrome are in fact females’ (p. 120). Barr body tests of skin samples were by then relatively easy and inexpensive, and Polani et al. presented their three case studies, reporting that they identified male ‘skin sex’ (that is, the absence of Barr bodies in skin samples) and concluded that ‘at least some patients with Turner’s syndrome and coarctation of the aorta have a chromosome pattern which is characteristically seen in males’ (Polani et al., 1954: 121).

In the case of Klinefelter’s syndrome, it was discovered in 1956 that some individuals with Klinefelter’s Syndrome were chromatin-positive; that is, a Barr body was identified and the individual was assumed to have an XX karyotype (Bradbury et al., 1956). After the buccal smear of one individual with a diagnosis of Klinefelter’s syndrome appeared to show that he was ‘a genetic female’ (p. 689), Bradbury et al. from the University Hospitals, Iowa, tested four previous patients with a diagnosis of Klinefelter’s syndrome and got similar results. They thus assumed that the patients had XX karyotypes. In April 1958, a symposium focused on sex differentiation and development in humans and animals was held at the Royal Society of Medicine in London, organized by the Society for Endocrinology. It aimed to report on the state of scientific knowledge in the UK and the rest of the world, and featured discussions of both Turner and Klinefelter’s syndromes. A paper on the ‘sex chromatin’ discussed contemporary testing procedures for identifying presence or absence of sex chromatins in cells. Chief among the reported applications for this testing, referred to as ‘nuclear sexing’, were the identification of intersex in early life and the ‘spectacular advances’ in the classifications of Turner and Klinefelter’s syndrome as ‘sex reversals’, referring to the ‘chromatin-negative (genetic-male) feminoids of Turner’s syndrome’ and the ‘chromatin-positive (genetic-female) masculinoids of Klinefelter’s syndrome’ (Lennox et al., 1958: 126–127).

During the 1950s, the diagnosis of Turner and Klinefelter’s syndromes shifted from describing a cluster of symptoms to enacting those described as examples of ‘sex-reversal’. The discovery of Barr bodies and associated karyotyping technologies thus allowed for new classifications of populations of individuals as well as new forms of intersex to be imagined and enacted, beyond those previously identified in clinical practice (see Miller, 2006). With the doubt that was cast upon the biological sex of individuals with Turner or Klinefelter’s syndrome came a similar doubt cast upon their gender and sexuality. Between 1956 and 1959 there were a number of investigations as to whether individuals with Klinefelter’s Syndrome were atypical in their psychological sex or sexual behaviour, including investigations into ‘transvestism’ (Smith and Davidson, 1958; Overzier, 1958; Walter and Braütigam, 1958) ‘homosexuality’ (Jackson et al., 1956) and aspects of ‘feminine psychology’ (Manthey, 1958). Publications in the UK in this period also placed Klinefelter’s Syndrome within a ‘spectrum of sex’ which included intersex variations, homosexuality and transvestism in a series of gradations between ‘normal female’ and ‘normal male’ (Armstrong, 1958).

## 1959: Beyond XX and XY

Despite the move towards sex chromosomes (as interpreted through Barr bodies) as the best marker of sex and the best grounding for a classification of intersex, the sex-reversal hypothesis was not universally accepted. In 1956 in an article in the *Lancet*, Polani presented 25 case studies of female individuals diagnosed with Turner syndrome. He reported that: ‘Twenty of the patients had chromatin-negative nuclei (genetic males by inference) and four had chromatin-positive nuclei (genetic females by inference)’ (Polani et al., 1956: 119). The paper emphasized that this was an inference and suggested other reasons for the results:

we may be dealing with persons who have an XO pattern of sex-chromosomes. Whether such a condition is possible in man is unknown, nor is it known what its effect might be on sex-determination or on the chromatin status of the nuclei of somatic cells (Polani et al., 1956: 119).

Support for this speculation would come in 1959, with the publication of two key articles that provided evidence for more sex chromosome constitutions than just XX or XY. First, British researchers published evidence that individuals diagnosed with Klinefelter’s syndrome could have an XXY karyotype, rather than the assumed XX (Jacobs and Strong, 1959). Later that year, researchers elsewhere in Britain also published evidence that some individuals with Turner Syndrome did not have an XY constitution but one X and no Y (XO) (Ford et al., 1959). This challenged the assumption that these syndromes were evidence of ‘sex reversals’ (and ended the use of language such as ‘feminoids’ and ‘masculinoids’). However, by this point both syndromes were firmly associated with classifications of intersex.

This history of sex chromosome variations intersects with Britain’s post-war investment in atomic energy research, a national atomic bomb project, and research into the biological and medical aspects of radiation, which was the responsibility of Britain’s Medical Research Council (MRC) (De Chadarevian, 2006: 710). British researcher Patricia Jacobs began working at a newly established unit of the MRC in Edinburgh in 1957, and soon after went to learn human chromosome techniques from Laszlo Lajtha in Oxford and Charles Ford at the Harwell MRC unit (Harper, 2006: 85). Jacobs returned to Edinburgh in 1958 and established new techniques of chromosome analysis of human bone marrow (e.g., Ford et al., 1958). Jacobs was studying chromosome damage in radiation-induced leukaemia when she identified an XXY karyotype in bone marrow samples from individuals with Klinefelter’s Syndrome (Harper, 2006: 84–86). The British government’s establishment of the MRC after the war enabled a professional network through which biological materials such as blood, skin and bone marrow could move. While directed towards atomic and radiation concerns, this network facilitated developments in sex chromosome science in the 1950s. The MRC unit was in a radiotherapy department, so Jacobs could access bone marrow from patients with leukaemia, but not necessarily radiation-induced leukaemia. Jacobs was therefore actively looking for research subjects, and as she reports, was ‘offered a Klinefelter patient’ by the endocrinologist John Strong (Harper, 2004). In Jacobs’ own account, from an interview in 2004, ‘everybody assumed that they were sex-reversed females, so everybody expected them to be XX, but I thought I could see 47 chromosomes. And I thought I could see something that was compatible with being a Y’ (Harper, 2006: 86).

The description of XXY karyotypes in some individuals diagnosed with Klinefelter’s syndrome was significant. Indeed, as Ursula Mittwoch (1967) emphasized, the Jacobs and Strong paper ‘provided the first concrete evidence that abnormalities of sexual development may be caused by an abnormal sex-chromosome constitution’ (p. 109). Earlier, Barr had suggested that Barr bodies could be a tool for aligning clinical practices concerning the normalization of bodies with the genetics of sex. 1959 saw a development of what Kraus (2000) calls the ‘tooling’ of sex as classifications based on variation from visible bodily norms became more closely aligned with variation from chromosomal norms. However, this development was accidental; as Jacobs recounts, chromosomal variations were only of interest as they related to radiation-induced leukemia (Harper, 2004). Polani recounts that after reading the paper by Ford et al. on studying human chromosomes in bone marrow, he sent Ford bone marrow samples from himself and from two patients diagnosed with Turner and Klinefelter’s syndromes (Harper, 2006: 82). He saw the results at the end of 1958: The sample from the individual diagnosed with Turner syndrome had 45 chromosomes and was considered XO, and the individual diagnosed with Klinefelter’s syndrome had 47 chromosomes and an apparent XXY karyotype. Polani, Ford et al. published ‘A sex chromosome anomaly in a case of gonadal dysgenesis (Turner’s syndrome)’ in the *Lancet* in 1959, just three months after the Jacobs and Strong paper on Klinefelter’s syndrome.

These ‘startling’ discoveries led to a proliferation of discoveries of chromosome ‘abnormalities’ or ‘aberrations’ soon after, including XXXY, XXXXY and mosaicism (Penrose, 1966). Turner and Klinefelter’s syndrome, previously drawn together as ‘sex-reversals’, were thus further associated, now classified together as chromosome abnormalities or aberrations. It is worth noting that the identification of chromosome variations beyond XX and XY did not lead to acceptance of variation; rather, variations were framed through a ‘normalizing judgement’ (Foucault, 1975) that confirmed the hyperbinary norm of XX/XY, and defined all deviation from such a norm as an aberration or abnormality.

This was not just a medical norm; the association of the X chromosome with female sex and femininity, and the association of the Y with male sex and masculinity led to research questioning whether individuals with chromosome variations would show variation in (among other things) gender roles. A striking example of this is the research into the XYY ‘supermale’ (Richardson, 2013: 84). The Y was now firmly established as active, determinative, and indeed *the* marker of male sex. Jacobs and other researchers within the growing field of human medical genetics thus turned their attention to the recently described XYY karyotype. Jacobs recounts reading a piece of research in 1960 that found an over-representation of XXY individuals in security facilities, and also noticed that ‘a very significant proportion were XYY’ (Harper, 2006: 89). She began a research programme and published her findings in *Nature* as ‘Aggressive behaviour, mental sub-normality and the XYY male’ (Jacobs et al., 1965). Following this paper, a number of others reported increased criminality, aggression or impulsiveness among XYY men. The connection of the Y chromosome with maleness and masculinity led to associations with what were considered masculine traits, including aggression. The associations were prevalent; indeed, Richardson’s (2013: 84) research suggests that ‘by 1970, nearly two hundred papers on the link between XYY and aggression had appeared in the scientific literature’.

Narratives that associated the extra Y with an extra and problematic dose of masculinity were so influential that even the observation that individuals with XXY karyotypes were also over-represented in the same facilities could not seriously challenge the hypothesis. As Richardson notes, this apparent contradiction of the XYY theory was resolved through the reproduction of cultural norms about the differences between men and women, interpreting any XXY criminality as resulting from ‘feminine ineffectiveness and passivity’ and any XYY criminality as resulting from ‘a masculine overactive personality’ (p. 95). Richardson describes XYY ‘supermale’ research as an ‘embarrassing episode in the history of genetics’ (p. 82); a large-scale study in 1976 found that there was ‘no evidence … that men with either of these sex chromosome complements are especially aggressive’, and that there was therefore no social benefit in screening for these chromosomal variations (Witkin et al., 1976). However, the association between XYY (and XXY) and criminality has persisted, with published research investigating the links appearing as late as 2012 (Stochholm et al., 2012).

The example of XYY ‘supermale’ research is informative. XYY would seem to fit the current DSD classification definition of ‘congenital conditions in which development of chromosomal, gonadal, or anatomical sex is atypical’ (Hughes et al., 2006: 554); however, XYY is not considered to be an intersex variation or a DSD. At this particular historical moment, XYY would also fit the ‘post-medicine’ definition of intersex offered by Miriam van der Have, as those individuals studied for their sex chromosome variation and their criminality could certainly have been described as experiencing ‘the socio-cultural consequences of being born with a body that does not fit with normative social constructions of male and female’ (Van der Have, 2016). Individuals with an XYY karyotype represent a population of individuals who, after 1959, came to be classified and defined by their chromosome variation. Despite the promise of knowing more about this population through genetic classification, this example reveals more about contemporary gender norms than it does about the population in question. The drive to ground classification in genetics did not elucidate XYY, but rather led to XYY being enacted as a reality that included assumed criminality through assumptions about masculinity. This episode is an interesting counterpoint to contemporary suggestions that future genetic science promises to sort out intersex classifications and provide better treatment and care.

## 1960s: Human medical genetics and intersex

The significant publications of 1959 and the first half of the 1960s established human medical genetics as an important field, compared to its previous status as a ‘sleepy subspecialty’ (Lindee, 2005: 1). Genetics began to be seen as offering the promise of practical advances in clinical medicine, which led to increasing numbers of practicing clinicians learning to work with chromosomes. This promise of genetics also had a significant influence on popular discourse. Lindee (2005) argues that this period saw the ‘realization of an idea … that all human disease is a genetic phenomenon subject to technological control’:

The idea that all disease is genetic disease is not an abstraction. It is a social experience manifest in language, technology, emotion, and policy. Increasingly, it plays a role in the legal system, in the practices of hospitals, in the research priorities of the armed forces, in the treatment of people with mental retardation, and in medical education. It has become an idea with social force. (p. 2)

XYY is a striking example of this, as social assumptions about masculinity and criminality came to define a population classified together through a shared karyotype. From 1959 through the 1960s, Turner and Klinefelter’s Syndrome also seemed to fit the pattern Lindee describes. Prior to the 1950s, a diagnosis of either Turner or Klinefelter’s syndrome was a description of a symptom cluster in one individual body. During the 1950s, this shifted, as the diagnosis began to mean ‘sex-reversal’ – a shift that enacted individuals in such a way that doubt was cast on their sex, gender and sexuality.

In the 1960s, Turner and Klinefelter’s syndromes were established as part of the pressing clinical concern over the ‘management’ of intersex variations. Against the background of this clinical concern and the growing field of human medical genetics, the 1960s saw the publication of key texts on intersex in Britain (e.g. Armstrong and Marshall, 1964; Ashley, 1962; Dewhurst and Gordon, 1969; Overzier, 1963). Most of these publications moved away from gonad-based classifications to ones based on karyotype, and all included Turner and Klinefelter’s syndromes. Dewhurst and Gordon’s *The Intersexual Disorders*, published in 1969, is a significant exception; whereas the other books published at this time gave sex chromosome variations precedence and thus centrality in the classification of intersex, Dewhurst assigned them a peripheral position. Turner and Klinefelter’s syndromes did get a chapter in the book: ‘Sex chromosome abnormalities’. However, this was a short chapter, mainly focussed on diagnostic criteria. Furthermore, this chapter came after a chapter on ‘Transvestism and transsexualism’ even though in a later interview Dewhurst would make it clear that he did not consider ‘transvestism’ or ‘transsexualism’ to be intersex variations, but rather included them to cover as wide an area as possible, and because they were often confused with intersex (King, 1980). Sex chromosome variations are discussed after this chapter, while still included in a taxonomy of ‘intersexual disorders’.

Dewhurst was an obstetrician and gynaecologist, and his interest was in ‘disorders’ that are obvious and visible at birth. His approach was similar to that of Money, whose work was becoming increasingly influential. For Dewhurst and Gordon (1969), chromosome variations were peripheral to the classification of intersex; the defining features of his intersex classification were genitals, and his concern was the need to assign gender as close as possible to the moment of birth. Coming at the end of 1960s, Dewhurst and Gordon’s book was influential – cited favourably by Money and Ehrhardt (1972) but cited more critically by recent intersex scholars and activists such as Fausto-Sterling (2000) and Morgan Holmes (2008).

With the development of human medical genetics as a field, chromosomes and karyotyping were central to many classifications of intersex. In clinical practice, however, chromosome variations were often peripheral within a ‘fuzzy’ system, where the central prototypical features were genitals and the central ambition the assignment of gender as close to the moment of birth as possible.

## The promises of genetics

The lack of consensus around the inclusion or exclusion of Turner and Klinefelter’s syndrome in intersex classifications continues. Some individuals with diagnoses of Klinefelter’s syndrome reject the inclusion of the syndrome within intersex, while others are more positive about it (Harper, 2007; see also Cameron, 1999). Individuals from Turner-syndrome support associations have rejected the classification of Turner syndrome within a taxonomy of intersex (Harper, 2007). These individuals have a stake in the classification of ‘their’ diagnosis. As Davis (2015) has shown, a lot depends on the acceptance or non-acceptance of the DSD terminology, including issues of self-perception, family relationships, and relationships with medical professionals. Classification matters to individuals, enacting their realities in significant ways. There is also seemingly no medical consensus about whether these syndromes should be included in the DSD classification. The European Society for Paediatric Endocrinology, which, along with the Lawson Wilkins Pediatric Endocrine Society, had co-sponsored the Chicago meeting that agreed on the DSD terminology in 2005, published a classification of paediatric endocrine diagnoses two years later (Wit et al., 2007). In this classification, Turner and Klinefelter’s syndromes were no longer defined as disorders of sex development, but rather as ‘chromosomal abnormalities’, a subcategory of ‘syndromes with endocrine features’ (Wit et al., 2007: 101). This was not the only example of professional disagreement about what exactly counts as a disorder of sex development, nor the only one that attempted to exclude Turner and Klinefelter’s syndromes from the classification. Writing in the *Journal of Pediatric Urology* in 2010, Aaronson and Aaronson (2010) argued that, while the term DSD had become quickly accepted in the medical community, the consensus statement ‘did not specify precisely which conditions should be considered under this heading’ (p. 444). They recommended that ‘the term DSD be strictly limited to those conditions traditionally regarded as intersex, and that these should be classified on the basis of their underlying gonadal histology’ (Aaronson and Aaronson, 2010: 444; see also Hughes, 2010a). For these researchers, Turner and Klinefelter’s syndromes should be considered ‘chromosome aberrations’, not classified within the ‘mainstream of intersexuality’ (p. 446). The suggestion of a mainstream of intersexuality is informative, and suggests a more ‘fuzzy’ classification system, with mainstream objects (fitting the prototype), and those on the periphery that are less typical of the category.

The debate within the medical profession about the categorical inclusion or exclusion of Turner and Klinefelter’s syndromes illustrates an ambiguity at the heart of the DSD classification system. The classification system is constructed differently depending upon whether Turner and Klinefelter’s syndromes are considered the ‘mainstream’ that is central to the DSD classification system, or peripheral and excludable. Indeed, Eric Vilain and David Sandberg (both present at the Chicago meeting) argued in 2009 that excluding Turner and Klinefelter’s syndromes from DSD ‘implies that atypical genital appearance is the sine qua non of DSD’ (Vilain and Sandberg, 2009: 9). For them, excluding Turner and Klinefelter’s syndromes from the DSD classification

implicitly undermines the value of the DSD nomenclature introduced in the consensus statement by weakening the inherent logic behind the classification system, which is about multiple aspects of sexual development, and not exclusively focused on the appearance of the genitals and the issue of gender assignment. (p. 10)

If Turner and Klinefelter’s syndromes are included as DSDs or intersex variations, estimates of frequency are necessarily higher than if they are not (as demonstrated by the debate between Fausto-Sterling and Sax). But if they are excluded it seems the very nature of the classification system changes, from a classification that is seemingly about multiple aspects of sexual development, to a classification that is focused on genitals and gender assignment. Accordingly, for Vilain and Sandberg, the very ‘value’ of the DSD nomenclature is undermined by the exclusion of Turner and Klinefelter’s syndromes. This is a particularly strong statement of the stakes for medicine in the inclusion or exclusion of these syndromes from classifications or taxonomies of intersex or DSD.

The DSD classification system is ‘fuzzier’ than it first appears. There is a certain incoherence or messiness within the logic of the consensus itself. After the ‘example of a DSD classification’, the statement suggests: ‘While consideration of karyotype is useful for classification, unnecessary reference to karyotype should be avoided; ideally, a system based on descriptive terms (for example, androgen insensitivity syndrome) should be used wherever possible’ (Hughes et al., 2006: 555). This is an intriguing statement, and it is unclear why a table sketching a classification based on karyotype should be followed by a statement that classifications in practice would ‘ideally’ avoid ‘unnecessary reference’ to the karyotype. As with the examples discussed by Bowker and Star (1999), the DSD classification system can be defined as both striving for an Aristotelian ideal that is free of ambiguity, while in practice being a fuzzier, more heterogeneous classification, structured around prototypes rather than essences. Yet while this raises social and ethical questions, it does not necessarily undermine the authority of DSD classifications within the medical profession. Communities of scientific practice have been shown to work with multiple and contradictory classification systems at the same time, often without conflict (Mol, 2002; Sommerlund, 2006).

The 2006 consensus statement accomplishes this, in part, through the implicit promise that future discoveries and techniques in genetic science will ‘sort out’ ambiguities, leading to clearer classifications, quicker diagnoses and better treatments. In fact, the ‘Future studies’ section of the statement begins with reference to two tables of genes ‘known to be involved in disorders of sex development’, urging further research in this area (Hughes et al., 2006: 559–560). The ten-year update goes further along this line, stating that future diagnostic approaches will use ‘next generation sequencing (whole exome or whole genome sequencing) as a first-line clinical test [that will] lead to a rapid and definitive diagnosis in the majority of cases’ (Lee et al., 2016: 167). Despite identifying obstacles both social and scientific, the article is optimistic that next generation gene sequencing will resolve many current ambiguities in diagnosis:

However, this approach is faced with hurdles including long turnaround times, high costs, a lack of insurance approval or national healthcare system coverage and difficulties in the interpretation of the results, such as questions about reporting of nonrelated incidental findings or sequence variations which are significant but not recognized as such. *These obstacles are likely to be overcome in the future*, and next-generation sequencing is likely to become one of the first methods used for the diagnosis of DSD. (Lee et al., 2016: 167, my emphasis)

Here, the promissory nature of genetics suggests that at some point in the future (social obstacles such as healthcare coverage and insurance notwithstanding) genetic science will facilitate clarity in intersex classifications, rapid and definitive diagnoses, and better treatments.

In both the 2006 consensus statement and the 2016 update, biomedical classifications and data are framed as promising more than psychosocial or cultural approaches. This is not only true of DSD, but of a recent interest in what attention to ‘rare diseases’ can reveal, both about these conditions and about non-affected populations. Indeed, iDSD, a contemporary international biomedical registry for intersex variations, is modelled on the rare disease approach, allowing medical professionals to access and study intersex biology on remote, despite perhaps never knowingly meeting an intersex person.

Hughes emphasized the fact that the 2006 consensus statement led to a focus on genetics when he identified the Chicago meeting as the ‘starting point’ that ‘spawned a classification system that recognised the impact molecular genetics could have, literally at the cot-side’ (Hughes, 2010b: 160). Compare this to the statement made by Barr in 1953, that karyotype-based classifications would be ‘the more valuable since the dominant sex could be detected *in infancy* by a relatively simple procedure’ (Moore et al., 1977 [1953]: 109, emphasis in original). iDSD (funded by the Medical Research Council 2011-2017) is the successor project of EuroDSD, funded by the European Union. This network enacts intersex variations/DSDs as rare genetic diseases, with the assumption that bodily difference is best understood through more and better, ongoing, future-focused genetic and molecular knowledge production. Once again, it remains to be seen whether this faith in increasing biomedicalization of the intersex experience will improve the health and lives of those affected.

Medical classifications are socially and historically situated, as well as determined by scientific and technological possibilities of the time. DSD is explicitly a future-oriented classification system, aiming to be ‘sufficiently flexible to incorporate new information but robust enough to maintain a consistent framework’ (Hughes et al., 2006: 554). But how much faith should people affected by these classifications put in the promise that, through this classification system and a grounding in a promissory genetic science, the future will do more justice to their lived experiences (medical and non-medical)? Through an historical view of early moments of promissory genetics, and indeed in the light of other studies on the future-orientation of biomedical science (e.g. Rose, 2007), one can see that the idea that obstacles both scientific and social ‘will likely be overcome’ at some point in the future and thus lead to better treatment is at least misguided. It is lacking in context, and insufficiently attentive to the ethical concerns of the present. It is also inattentive to how DSD enacts the realities of those classified.

## Conclusion: Resistance

Many intersex activist individuals and organisations have condemned (and continue to condemn) the move to the DSD classification system. This is activism in the face of widespread acceptance of DSD in the medical community (Aaronson and Aaronson, 2010; Hughes, 2010a). Indeed, the DSD classification system has been described as having ‘arrived at the high altar of medical practice’ (Hughes, 2010b: 161). However, Bowker and Star (1999) remind us that there is always room for resistance in classification systems (p. 49). Early intersex activism in the 1990s used a variety of approaches, including satire and irony, to resist medical authority and dominance. ‘The murk manual’ was a satirical guide to understanding medical writing on intersex, published in 1997 in a special issue of the journal *Chrysalis*, edited by intersex activists Cheryl Chase and Martha Coventry. In this ironic glossary, Klinefelter’s syndrome is defined: ‘One of that large class of syndromes named for people who did not have the syndromes. Even Lou Gehrig’s Disease has been renamed for some doctor who probably couldn’t even hold a bat’ (Carter, 1997: 10). Naming and classifying syndromes matters. Here, Klinefelter’s syndrome is reclassified as belonging to a larger class of syndromes, not defined by symptom clusters or bodily variations, but by the process of naming and classification that confers authority onto the medical profession while silencing the individual given the diagnosis. This satirical shift emphasizes the stakes involved for individuals in biomedical classifications, by drawing attention to the power of naming, and the silencing of intersex people through the privileging of a biomedical narrative. This ironic classification draws attention to the fact that classification-as-practice can confer and deny power and authority, blocking democratic participation in biomedical science. It also suggests that resistance may be possible.

Earlier I discussed another example of resistance, the creation of a different classification that is not structured around genitals, gender assignment or even the ‘truth’ of sex to be found in gonads or chromosomes, but instead in the social consequences of biomedical classifications. This ‘post-medicine definition’ defines intersex as ‘the lived experience of the socio-cultural consequences of being born with a body that does not fit with normative social constructions of male and female’ (Van der Have, 2016). Bowker and Star (1999) hold that ‘[t]he only good classification is a living classification’ (p. 326), and the ‘post-medicine’ understanding of intersex is a living classification. By focusing on ‘lived experience’ and ‘socio-cultural consequences’ rather than specific bodily differences, this classification subverts the medicalization of intersex, and indeed makes room for what intersex activists have long argued, that the over-medicalization of variations is harmful, but the variations themselves are not. The body in this classification is enacted as one that has been acted upon and must be lived with. This definition of intersex looks to a radically different future from the future of promissory genetics in the DSD classification system. The future suggested by this social definition is one where normative social constructions, biomedical practices and the socio-cultural consequences are not fixed, but open to change. Lived experience also allows for self-determination within the classification system. It is possible within this definition for individuals with a diagnosis of Turner or Klinefelter’s syndrome to self-identify with the classification, or not; the authority of classification is subverted.

Classifications are always historically and socially situated, which allows for subversive reimaginings of medical classifications. Resistance can also take the form of new and different orders of classification, for example those that prioritise the lived experience and shifting social consequences of being classified. The 1950s provided an example of a previous attempt to ground the difficult business of sorting out intersex classifications in genetics (at the level of the chromosome). However, grounding intersex in chromosomes in the 1950s did not improve clinical care for individuals with variations of their sex characteristics. The current DSD classification system is insufficiently attentive to the social and ethical needs of the present and relies on a vague promise that, sometime in the future, classification, diagnosis, and clinical practice will be sorted out for the benefit of both the medical profession and for individuals affected by classification. The history of the shifting understandings of Turner and Klinefelter’s syndromes illustrates that this faith in promissory genetic classifications may be misplaced. Instead, resistant social and historical classifications (always living) of bodily difference and the situated experience of the medicalization of difference may be more useful to do justice to the historical and contemporary lived experience of those affected by classifications of intersex.

## Notes

1. For a history of essentialist philosophy beyond Aristotle and its relevance to intersex, see Delimata (2016).

2. For a brief history of the relevance of mouse models to the development of theories of sex development in mammals beyond *Drosophila* see Goodfellow and Darling (1988).

3. John Money et al. at Johns Hopkins promoted a ‘best sex’ approach which argued that gender identity was malleable until approximately eighteen months (Kessler, 1990: 6). That is, a child could be successfully raised as either a boy or a girl, as long as their genitals are surgically and hormonally made to correspond with assigned gender, and no doubt is communicated to parents of the child as to their gender assignment (Kessler, 1990: 7). Within this system ‘true sex’ (whether located in gonads or chromosomes) was less important than the gender of rearing and aesthetics of the genitals. Money’s influence is rightly emphasised in intersex history; it has been argued that his work from this period constituted ‘the essential writing on the subject’ until the 1990s when the Intersex Society of North America was founded, and scholars such as Kessler and Fausto-Sterling began to publish on intersex (Reis, 2009: 116–117).
